# Correction to “HLA‐B Associates With Distinct Disease Expression in Juvenile Spondyloarthritis: A Retrospective Cohort Study”

**DOI:** 10.1002/acr2.90158

**Published:** 2026-09-15

Bikas, D.V. , Brandon, T.G. , Newby, B.N. and Weiss, P.F. (2026), HLA‐B Associates With Distinct Disease Expression in Juvenile Spondyloarthritis: A Retrospective Cohort Study. ACR Open Rheumatology, 8: e90122. 10.1002/acr2.90122 42610327 PMC13482771

Following publication, we identified a typesetting error in Table 2 of the “Results” section. This error was not present in the revised manuscript submitted by the authors and was unfortunately missed during proof review.

In Table 2, the superscript “c” was incorrectly assigned. The superscript should be removed from the value at the intersection of B*18:01 and **IBD‐AA** and reassigned to the value at the intersection of B*35:02 and **IBD‐AA**.

We apologize for this error.

